# The microbiome of draught no- and low-alcohol beer

**DOI:** 10.3389/fmicb.2026.1966818

**Published:** 2026-10-28

**Authors:** Giulia E. Roselli, Katherine A. Smart, Matthew Crow, Chris D. Powell

**Affiliations:** 1 Division of Microbiology, Biotechnology and Brewing Science, School of Biosciences, University of Nottingham, Loughborough, United Kingdom; 2 Diageo Supply Chain and Procurement, International Technical Center, Menstrie, United Kingdom

**Keywords:** alcohol-free, draught, NoLo, non-alcoholic beer, spoilage, third-generation sequencing

## Abstract

The microbiological ecosystem of commercially available draught dispensed no- and low-alcohol beers was evaluated, with a focus on identifying predominant microorganisms with spoilage potential and characterizing variation in community structure. More than 50 samples, representing 20 no- and low- alcohol beers (7 different beer styles: lager, India pale ale, pale ale, wheat, radler, pilsner and amber ale; and 17 brands) were collected for analysis across 12 different public houses (accounts) in Nottingham, UK. Bacterial and fungal identification was achieved via third generation sequencing technologies, using amplicon-based nanopore sequencing (Oxford Nanopore Technologies). Analysis of samples indicated that species of *Acetobacter, Brettanomyces* and *Saccharomyces* were core microbiome members. The account from which a beer was sampled was shown to harbor distinct microbial communities and was the dominant factor in determining microbial community structure, explaining 72.6% of variation. This is the first study to apply third-generation sequencing technology for the analysis of draught no- and low-alcohol beers. The findings underscore the vulnerability of draught products to microbial spoilage, and highlight the importance of implementing strict hygiene control focused on draught lines, taps, keg couplers and nozzles, especially when dispensing microbiologically sensitive no- and low-alcohol beverages.

## 1 Introduction

Traditionally beer has been regarded as a microbiologically stable product, due to innate characteristics that act to inhibit the growth of microbes. These include a lack of nutrients, the presence of hop bitter acids (approx. 17–55 ppm iso-alpha acids), low pH (approx. 3.9–4.4), low oxygen (< 0.1 ppm), elevated concentrations of carbon dioxide (2–2.5 v/v) and the presence of ethanol (4–8 % ABV) (Leistner, 2000; Menz et al., 2009). However, the alcoholic beverage sector is currently experiencing growth in demand for no- and low-alcohol (NoLo) variations of standard product types. In no- and low-alcohol beers^1^, the loss or reduction of ethanol combined with the increased availability of fermentable sugars weakens the antimicrobial system, acting to alter the microbial niche (Quain, 2021; Roselli et al., 2024). This renders the product inherently more prone to microbial spoilage, permitting the survival and growth of a range of microorganisms, including those not typically associated with beer (Bartlett, 2023; Maust et al., 2026; Rachon et al., 2026; Roselli et al., 2026, 2024).

In brewing, NoLo beers are typically manufactured in the same facility as standard beer types, using broadly the same ingredients and process steps. Conventionally, microbiological control occurs within the brewery, through the adoption of closed vessels and efficient cleaning-in-place (CiP) systems. Production stages such as wort boiling, filtration, sterile filling and pasteurization of final products also form a key part of microbial assurance. Due also to routine microbiological quality control checks, packaged beers in cans, bottles or kegs are typically found to be ‘commercially sterile' with microbial loads below 1 CFU/L, and spoilage is rare (Roselli et al., 2026). At the current time the majority of NoLo beers are sold predominantly in small pack form (cans or bottles), however, consumer demand for these beverages on draught is increasing (Quain, 2021; Roselli et al., 2026). This poses a challenge to the industry since in many countries (including the UK), draught beer is often dispensed using ‘long draw' or ‘long line' systems which are maintained and managed by individual proprietors and fall outside the control of the brewer. Draught dispense systems have been recognized as a source of spoilage for several decades, with early reports dating back to the 1980s (Casson, 1985; Harper, 1981; Harper et al., 1980). These continue to be documented in more recent studies that have shown a close association between dispense systems and the presence of planktonic microbes and biofilms (Jevons and Quain, 2021, 2022; Quain, 2015; Walker et al., 2007). The formation and persistence of biofilms is largely explained by the environmental conditions present within long-line dispense systems. For example, there are opportunities for oxygen ingress and temperature fluctuation which, along with the presence of beer residues, can be conducive to microbial growth. Dispense equipment also contain locations for microbial attachment and retention, particularly when low throughput or suboptimal cleaning regimes are applied (Jevons and Quain, 2021). Under these conditions, microorganisms are able to develop structured biofilms that are more resistant to routine cleaning and from which microorganisms can periodically slough and contaminate the beer (Quain, 2025). The inherent vulnerability of no- and low-alcohol beers is therefore amplified at the point of dispense, as it can become contaminated with any yeast or bacteria that are present as it passes through the dispense system (Quain, 2021). Consistent with this, previous work has demonstrated that more than half of no- and low-alcohol beers sampled on draught were of ‘unacceptable' quality due to the presence of microbial growth (Roselli et al., 2026). Specifically, microbial contamination can lead to spoilage of draught beer, manifesting as sensorial defects ranging from subtle flavor changes to pronounced alterations in flavor, aroma and appearance (Quain, 2021; Vaughan et al., 2005). Previous investigations of the draught beer microbiome have largely focused on the detection of known spoilage microorganisms in standard-strength products (Bose et al., 2021; Casson, 1985; Harper, 1981; Harper et al., 1980; Hemmons, 1954; Hough et al., 1976; Jevons and Quain, 2022; Wiles, 1950). In contrast, relatively little work has explored the broader microbial ecosystem of no- and low-alcohol beers. Therefore, an improved understanding of these communities may help provide a more complete assessment of the stability and safety of NoLo beer types.

In order to capture the full breadth of microbial diversity in complex and variable systems, approaches beyond traditional culture-based methods have proven useful. Next-generation and third-generation sequencing (NGS/TGS) technologies have enabled comprehensive profiling of microbial communities across various environments, including soil (Kurokochi et al., 2021; McCann et al., 2016), seawater (Liem et al., 2021) and a range of food systems (Arikan et al., 2020; Chen et al., 2021; Ho et al., 2020; Murugesan et al., 2018). These technologies overcome the limitations of selective culturing, offering improved sensitivity, throughput and resolution (Bokulich et al., 2016). Among TGS platforms, the MinION nanopore sequencer (Oxford Nanopore Technologies, ONT) has recently been validated for the identification of beer-spoilage bacteria and yeast in brewery settings (Kurniawan et al., 2021; Shinohara et al., 2021). Compared with short-read technologies, MinION generates longer sequence reads that facilitate accurate bacterial taxonomic resolution through full-length analysis of the 16S rRNA gene (Yarza et al., 2008, 2014), while yeast identification can be enhanced through sequencing of the internal transcribed spacer (ITS) region and D1/D2 region of the 26S rDNA gene (Kurtzman, 2015). Sequencing-based microbiome data can be used not only to identify microorganisms present in a system, but also to compare microbial community structure at both the within-sample and between-sample level. This is commonly achieved using alpha and beta diversity metrics. Alpha diversity metrics describe the structure of a microbial community within an individual sample with respect to its richness (number of taxonomic groups), evenness (distribution of abundances of the groups), or both (Willis, 2019). In contrast, beta diversity metrics describe differences in community composition between samples, using either abundance-based or presence/absence based measures (Kers and Saccenti, 2022).

Previously, we conducted a survey to examine the quality of NoLo beers dispensed using draught systems (Roselli et al., 2026). In the current study, the same samples were evaluated for microbial composition and diversity. Here, we report the first application of third-generation based sequencing to analyse the microbiome of draught no-and low-alcohol beers, enabling both quantitative and qualitative assessment of the microbial communities. By combining taxonomic profiling with alpha and beta diversity analyses, the aim of this study was to assess the microbial communities present in draught NoLo products and to identify key factors that explain the variation in community structure.

## 2 Materials and methods

### 2.1 Beer samples and accounts

Commercially available no- and low-alcohol beers (*n* = 20, Table 1) were purchased over a period of three months from January to April 2025. Specific beer brands and the licensed on-trade premises (‘accounts') included in this study are anonymised and consistent with previous work (Roselli et al., 2026). Across 20 different beer products a total of 49 samples were collected, representing 7 different beer styles, 17 brands and 12 different accounts. The beers sampled were either non-alcoholic or low-alcohol beers with a reported ethanol content ranging from 0.0 – 0.5% ABV (% v/v). Beer styles included “lager” (L), “India pale ale” (IPA), “pale ale” (PA), “pilsner” (P), “amber ale” (A), “radler” (R) and “wheat” (W). Beers within the stout category were not subject to investigation during this study.

**Table 1:** No- and low-alcohol beer samples collected for comprehensive study.

Sample	(*n*)	Account	Beer style	ABV (%)^*^	Production method	[Initial DNA] ng/μL	[Fungal DNA] amplicon	[Bacterial DNA] amplicon
L1A	2	5	Lager	0.5	Physical	27.5	346.5	164.0
L1B	2	6				15.8	282.5	260.5
L2	3	3	Lager	0.5	Biological	24.0	264.5	242.0
L3	3	12	Lager	0.0	Physical	43.0	300.0	325.0
L4	3	11	Lager	0.0	Physical	11.0	327.5	396.5
L5	2	4	Lager	0.5	Physical	14.7	331.5	530.5
IPA6	3	3	Indian Pale Ale	0.5	Biological	20.5	275.0	264.0
IPA7	3	2	Indian Pale Ale	0.5	Biological	14.6	407.5	377.0
IPA8	2	7	Indian Pale Ale	0.5	Biological	26.5	326.0	361.5
IPA9	2	4	Indian Pale Ale	0.5	Biological	29.0	381.0	222.5
IPA10	1	6	Indian Pale Ale	0.5	Biological	32.5	333.5	277.0
IPA11	3	7	West Coast IPA	0.5	Physical	14.4	328.5	454.0
PA12	3	9	Pale Ale	0.5	Physical	85.0	288.0	306.0
PA13	2	1	Pale Ale	0.5	Biological	9.2	375.0	459.5
PA14	3	1	Pale Ale (Hazy)	0.5	Biological	35.5	348.0	317.5
PA15	1	4	Pale Ale	0.3	Biological	8.0	358.5	325.5
PA16	2	4	Pale Ale	0.5	Biological	13.0	331.5	337.0
A17A	2	7	Amber Ale	0.5	Biological	11.4	283.0	293.5
A17B	1	10				17.0	318.0	307.5
P18	2	4	West Coast Pilsner	0.5	Biological	87.5	363.5	329.5
R19	3	4	Radler	0.5	Physical	17.0	316.0	283.0
W20	3	8	Wheat	0.0	Physical	11.9	540.0	341.5

^*^As reported on product labels.

### 2.2 Study design and sample collection

Sampling was conducted followed the protocol of Mallett and Quain (2019), designed to minimize the effect of uncontrollable factors known to influence draught beer quality such as the frequency and time of line cleaning, and variation in beer throughput. Replicate samples consisted of either repeated pours of the same product from the same account on separate days, or the same product collected from different accounts.

Draught beers were purchased from each account and approximately 200 mL was transferred directly into sterile Duran (250 mL) bottles. Samples were transported cold and stored overnight at 4 °C prior to processing. All subsequent steps were performed aseptically. To promote the growth of microorganisms present, samples were incubated statically at 30 °C for 96 h (‘forced'), with loosened caps to enable gas exchange (Mallett et al., 2018). Following incubation, 50 mL of each sample was centrifuged (3, 220 x *g*, 20 mins, 4 °C) and the supernatant was used in a linear study (Roselli et al., 2026). The pellet(s) were washed twice in 20 mL PBS (phosphate buffered saline, Merck, Germany) and stored frozen (-20 °C) until further analysis.

### 2.3 DNA extraction

Pellets from replicate samples of the same product and account were pooled prior to extraction, whereas samples representing the same product collected from different accounts were instead processed individually and differentiated using an alphabetical suffix (i.e. L1A/L1B and 17A/17B; Table 1).

DNA extraction was performed using the *Quick*-DNA^TM^ Fecal/Soil Microbe Miniprep Kit (Zymo Research Corporation) according to the manufacturer's protocol, with minor modifications to the mechanical lysis step, as described here. Pellets of 50–100 mg (wet weight) were resuspended in 150 μL of PBS and transferred to a ZR BashingBead^TM^ Lysis Tube containing 750 μL BashingBead^TM^ Buffer. Mechanical lysis was performed using a MagNA Lyser (F. Hoffmann-La Roche Ltd). Samples were processed for five cycles at 7,000 rpm for 1 min with a 3 min rest between cycles, giving a total active bead-beating time of 5 min. DNA was then purified according to the remaining steps of the manufacturer's protocol. Extracted DNA concentration was determined using a NanoDrop 2000 spectrophotometer (Thermo Fisher Scientific).

### 2.4 PCR amplification of fungal ITS regions and bacterial 16S rRNA genes

Yeast genomic DNA was amplified using the high-coverage SSU515Fngs-F and LR5-R primer pair (Table 2) targeting the ITS region, as described by D'Andreano et al. (2021). Bacterial 16S rRNA genes were amplified using primers F27 and R1492 (Table 2). PCR reactions were prepared in 25 μL volumes containing 2 μL of DNA template, 12.5 μL of Q5^®^ Hot Start High-Fidelity 2X Master Mix, 1.25 μL of forward primer (10 μM) and 1.25 μL of the reverse primer (10 μM) and ultra-pure H_2_O to 25 μL. DNA amplification was performed using a Techne 3Prime PCR thermocycler with the following programme: initial denaturation at 98 °C for 30s; 35 cycles of 98 °C for 10s, 62 °C (ITS) or 66 °C (16S rRNA) for 30s (temperatures determined using NEB Tm calculator https://tmcalculator.neb.com/), and 72 °C for 20-30 s/kb; followed by a final extension at 72 °C for 2 mins. PCR products were cleaned using the Monarch^®^ PCR & DNA Cleanup Kit (New England Biolabs, Inc) and stored in 20 μL Monarch^®^ DNA elution buffer. Amplicon integrity and size were checked by agarose gel electrophoresis (Figure 1). The concentration of each of the 44 amplicons was measured using a Qubit 4 Fluorometer (Thermo Fisher Scientific) and the Qubit 1X dsDNA HS Assay Kit (Thermo Fisher Scientific; Q33231).

**Table 2:** PCR primers used to amplify fungal and bacterial rRNA genes.

Primer	Sequence (5'–3')	Target	Amplicon size (kb)	Reference
SSU515Fngs-F	-GCCAGCAACCGCGGTAA-	V1 region of 18S RNA – D12 region of 28S RNA gene	~ 3.5	D'Andreano et al., 2021
LR5-R	-TCCTGAGGGAAACTTCG-			
27F	-AGAGTTTGATCCTGGCTCAG-	Bacterial 16S rRNA gene	~ 1.5	Heuer et al., 1997
1492R	-TACGGYTACCTTGTTACGACTT-			

**Figure 1:**
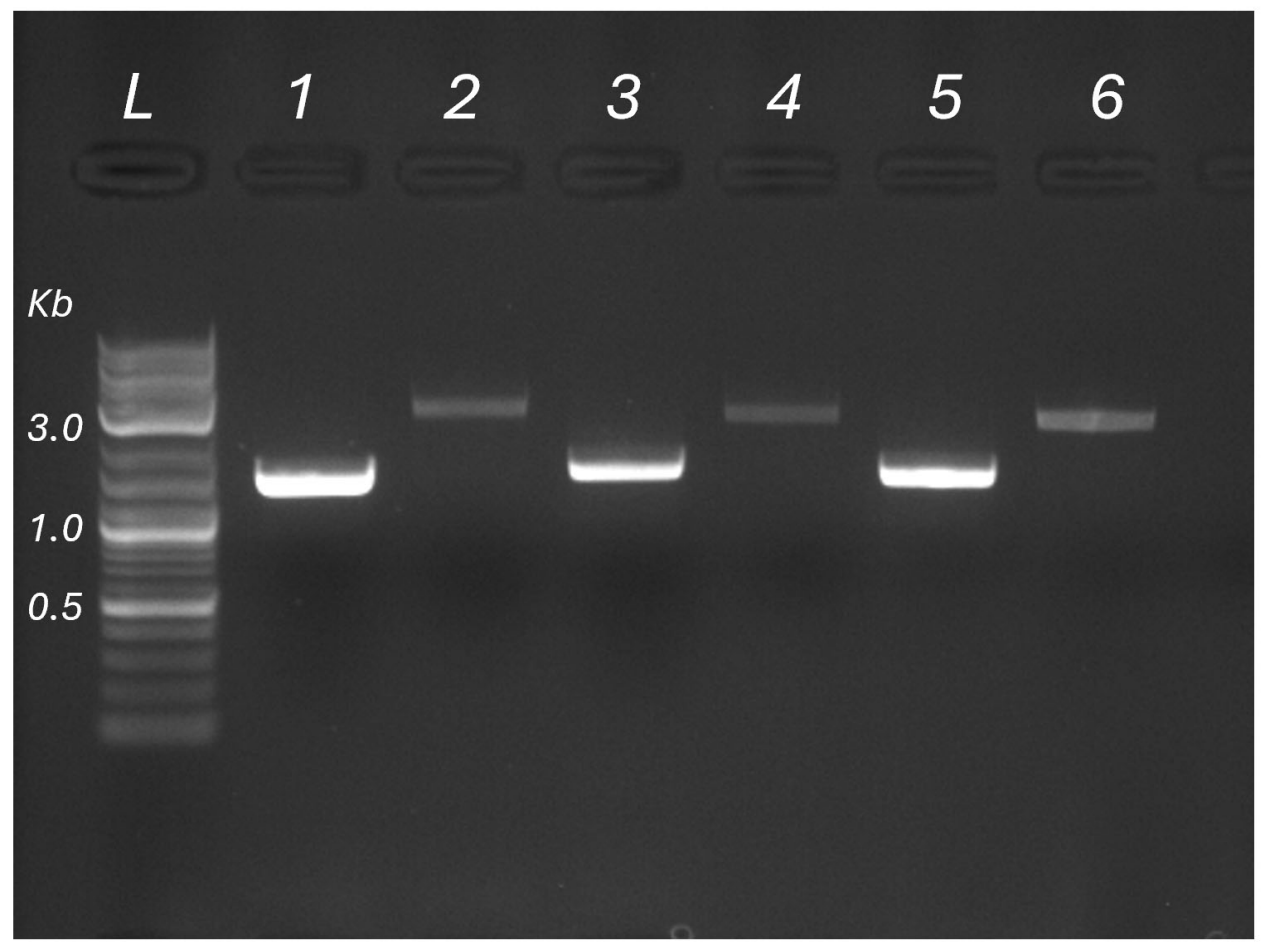
Representative analysis of bacterial (Ba ~ 1.5 kb) and fungal (Fu ~ 3.5 kb) DNA, amplified using 16S rRNA and ITS PCR respectively. *L:* Quick load 1 kb DNA ladder, *1* and *2:* Ba/Fu amplicons (sample L4), *3* and *4:* Ba/Fu amplicons (sample W20) and *5* and *6:* Ba/Fu amplicons (sample A17A). Comparable band patterns were observed across all analyses (data not shown).

### 2.5 DNA library preparation and MinION sequencing

Nanopore long read sequencing was performed in conjunction with DeepSeq (Next Generation Sequencing Facility, Center for Genetics and Genomics, The University of Nottingham, UK). The two amplicons for each sample were pooled together in roughly equimolar amounts. For most samples this involved combining 15 ng of the 16S amplicon (~ 1.5 kb) and 30 ng of the fungal ITS amplicon (~ 3–3.5 kb). Where amplicons did not meet this concentration threshold, lower amounts of 5 ng (16S) and 10 ng (fungal ITS) were collected and data was normalized to account for the variation.

A barcoded sequencing library was prepared for each amplicon pool using the Ligation Sequencing Amplicons - Native Barcoding Kit 24 V14 protocol (Oxford Nanopore Technologies; Version: NBA_9168_v114_revG_15Sep2022). This protocol used the Native Barcoding Kit 24 V14 (Oxford Nanopore Technologies; SQK-NBD114.24). Barcoded libraries were pooled to normalize the two different input amounts prior to the final sequencing-adapter ligation step. All purification steps were performed using AMPure XP beads (Beckman Coulter; A63882). The barcoded library preparation was loaded onto a PromethION R10.4.1M flow cell (Oxford Nanopore Technologies; FLO-PRO114M) and run on the PromethION 24 platform. Default sequencing parameters were used apart from barcoding, which was set to the higher stringency “barcode both ends” setting. This requires a barcode sequence to be recognized at both ends of the read in order for it to be categorized. Basecalling was performed using MinKNOW version 25.03.7, with high-accuracy basecalling model and read filtering of min_score = 9.

### 2.6 Sequence data analysis

Sequence data (FASTQ files) were analyzed using the cloud-based analysis platform EPI2ME^TM^ wf-metagenomics nextflow workflow (Oxford Nanopore Technologies, UK). Taxonomic classification was performed using the kraaken2 (k-mer based) classifier approach and the NCBI 16S_18S_28S_ ITS database set (https://www.ncbi.nlm.nih.gov/refseq/targetedloci/). Reads with Q-scores below 10 were excluded from analysis. The total number of reads generated, reads classified, correctly classified genus reads, and correctly classified species reads were recorded (Supplementary Table 1). The raw sequencing data generated in this study have been deposited in the European Nucleotide Archive (ENA) under accession number PRJEB123733.

### 2.7 Statistical analysis

Statistical analysis and data visualization were performed in R version (4.5.1) (R Core Team, 2025) in RStudio (version 2025.09.2). Data manipulation and visualization used the “tidyverse” (Wickham et al., 2019), “ggplot2” (Wickham, 2016) and “ggpubr” (Kassambara, 2022) packages. Microbiome data handling and alpha diversity analysis were performed using the “phyloseq” (McMurdie and Holmes, 2013) package. Statistical significance of these metrics was evaluated using the Kruskal-Wallis test. Beta diversity was assessed using Bray-Curtis dissimilarity via the “vegan” package (Oksanen et al., 2016). Community patterns were visualized using principal coordinates analysis (PCoA). Permutational ANOVA (PERMANOVA) was used to test whether any variables, including the concentration of ethanol, beer style, production method and/or account could explain any differences in microbial community composition.

## 3 Results

### 3.1 Sequencing analysis

Successful amplification of DNA from all samples was confirmed prior to sequencing (Figure 1), with clear and consistent bands observed for the bacterial (~ 1.5 kb) and fungal (~ 3.5 kb) targets. This verification step ensured that only robust amplicons were used for library preparation and subsequent sequencing. The library was sequenced and high-accuracy (HAC) basecalling was performed in real time using MinKNOW (version 25.03.7). More than 200,000 reads were generated for each amplicon pool in the length range of (110 – 11500 bp) with a quality score > 9, that were subsequently classified to their taxonomic group with EPI2ME filtering for reads with quality scores > 10. The high-read depth and consistent amplicon quality provided sufficient resolution for downstream taxonomic and diversity analyses. This, and other information regarding the quality of reads and assembly can be found in detail in Supplementary Table 1.

### 3.2 Taxonomic composition of draught no- and low-alcohol beer microbiomes

#### 3.2.1 Sample-level variability

In this study, the microbiome of commercially available draught no- and low-alcohol beers in the on-trade was analyzed using nanopore sequencing, with a focus on identifying potential spoilage microorganisms. To achieve this, a library of 16S rRNA bacterial and fungal ITS gene amplicons was generated using ONT Native Barcoding. Samples were obtained from 12 different trade premises (accounts) and a total of seven phyla, 87 genera and 207 species were detected. No pathogenic microorganisms (including *Escherichia coli* and *Salmonella* species) were detected in any of the samples collected. Analysis of the distribution of organisms was interpreted using a prevalence-abundance plot (Figure 2A) that revealed clear groupings of microbial genera across draught no- and low-alcohol beer samples. A small number of genera occurred both frequently and at high abundance (upper-right region), representing a core microbiome adapted to the environment of long-line dispense systems. In contrast, several genera appeared only sporadically but at relatively high abundance in isolated samples (mid-left region), suggesting sample-specific contamination events. Most genera were detected infrequently and at low abundance (lower-left region), likely reflecting rare or transient taxa introduced through handling or exposure at the point of dispense. Finally, a small number of genera were detected frequently but at low abundance (lower-middle/right region), indicating persistent minor community members, that are likely to play a passive or background role within the draught microbiome. Together, these patterns highlight the coexistence of prevalent spoilage-associated genera with a wider pool of low-prevalence, sample- or venue-specific microorganisms.

**Figure 2:**
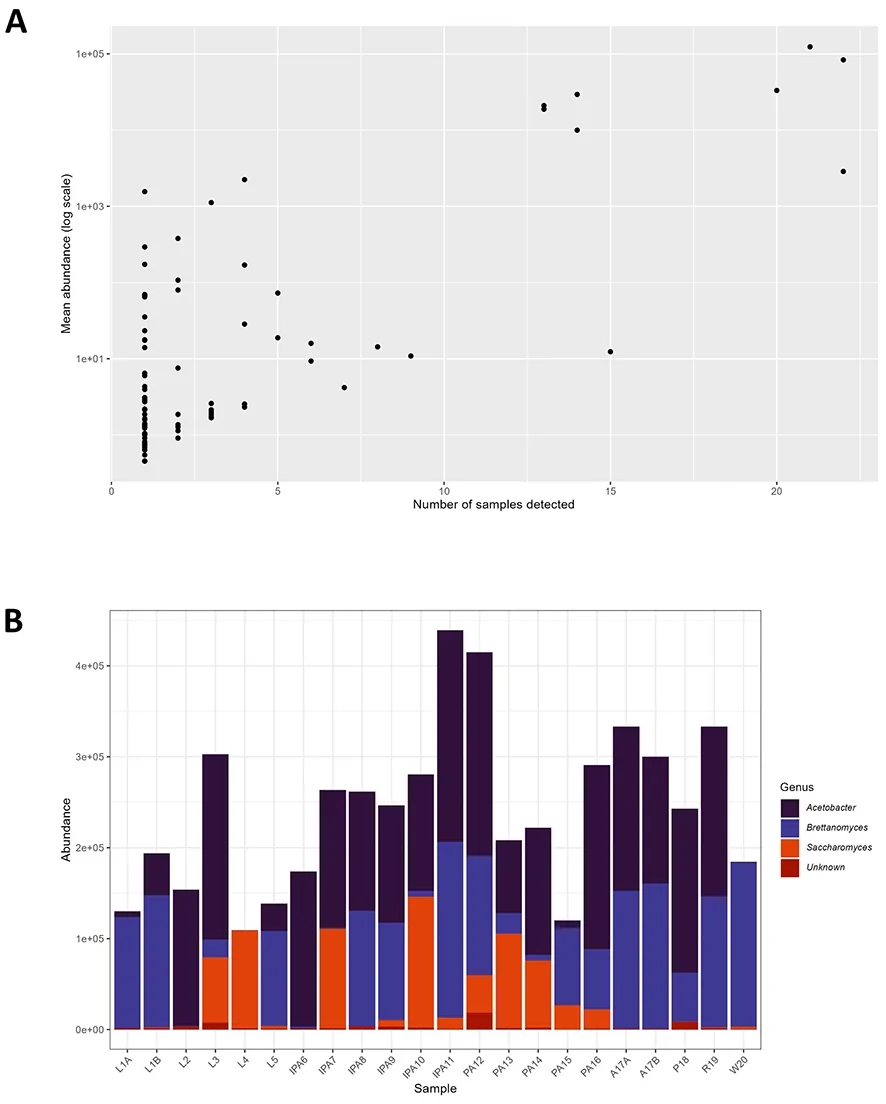
Microbial prevalence and abundance patterns across draught no- and low-alcohol beer samples. **(A)** Prevalence vs mean abundance scatter plot of microbial genera across samples. Individual dots represent individual genera. **(B)** Core microbiome of genera consistently present across ≥ 70% of samples.

The overall community was dominated by core microbiome members *Acetobacter, Brettanomyces* and *Saccharomyces*, all of which were consistently present across more than 70% of draught beer samples (Figure 2B). It should be noted that an unclassified genus-level group was also identified as part of the microbiome, likely reflecting non-specific or unassigned reads. Since these reads were low in relative abundance and lacked reliable genus-level classification, they were interpreted cautiously and were not considered to be a major biologically meaningful component of the core community. To further characterize the taxonomic composition across draught samples, the relative taxonomic abundances of the top twenty classified reads of draught no- and low-alcohol beer samples at both the genus (Figure 3) and species level (Figure 4) were analyzed. Genus-level patterns provided an overview of dominant microbial groups, while species-level profiles were able to distinguish which taxa were responsible for these broader community signatures. Sample-to-sample variability was evident at both the genus (Figure 3B) and species (Figure 4B) level. Some samples were dominated by few genera such as *Gluconobacter, Ogataea* (basionym: *Hansenula* (Yamada et al., 1994)) and *Saccharomyces (*L4), or *Brettanomyces* and *Levilactobacillus* (W20). Whilst others (L3, IPA9, and PA15) contained more mixed communities comprising multiple spoilage-relevant genera. Species-level data confirmed these patterns; for example, the dominance of *Brettanomyces* in W20 corresponded specifically to elevated relative abundance of *B. anomalus* and *B. bruxellensis* whilst *Saccharomyces-*dominated samples (L4, IPA7, IPA10, PA13 and PA14) contained isolates belonging to both *S. cerevisiae* and *S. uvarum*. Similarly, samples with high *Acetobacter* loads (L2, L3, IPA6, IPA7, IPA8, IPA9, IPA10, PA11, PA12, PA13, PA14, PA16, A17, P18 and R19), were typically driven by one or two species, most commonly *A. oryzoeni* and *A. sicerae*. However other species of *Acetobacter* were also present including, but not limited to, *A. indonesiensis*, A. *nitrogenifigens, A. orientalis, A. syzygii*, and *A. thailandicus*.

**Figure 3:**
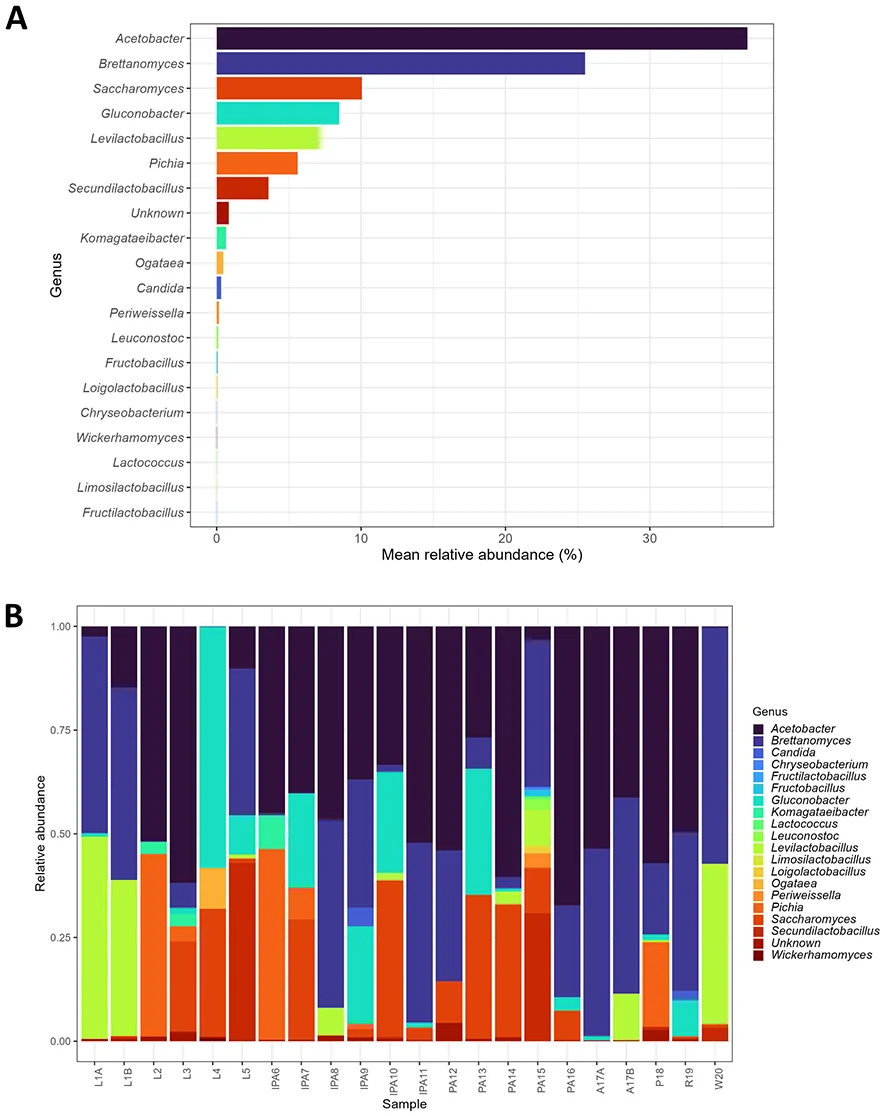
Relative taxonomic abundance (%) of top twenty genera of classified reads of draught no- and low-alcohol beer samples. **(A)** Mean relative abundance of top twenty genera of pooled samples. **(B)** Relative abundance profiles of top twenty genera of individual samples. Where samples are labeled ‘A' or ‘B', this indicates that they are the same beer type sampled from different accounts.

**Figure 4:**
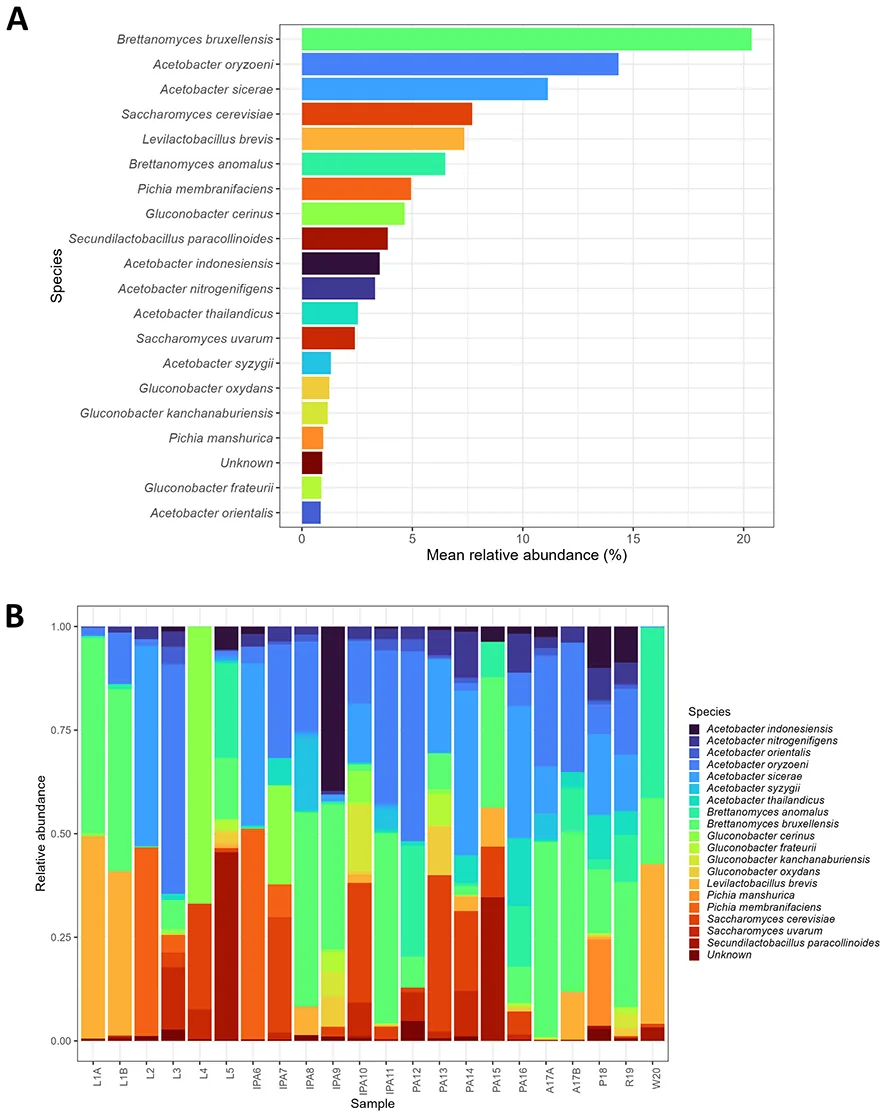
Relative taxonomic abundance (%) of top twenty species of classified reads of draught no- and low-alcohol beer samples. **(A**) Mean relative abundance of top twenty species of pooled samples. **(B)** Relative abundance profiles of top twenty species of individual samples. Where samples are labeled ‘A' or ‘B', this indicates that they are the same beer type sampled from different accounts.

#### 3.2.2 Account-level variability

Aggregating genus-level relative abundances by account revealed patterns that were not evident in the sample-level profiles, highlighting clear differences in microbial composition between trade outlets (Figure 5). Across most accounts *Acetobacter, Brettanomyces* and *Saccharomyces* were the dominant genera, with the exception of account 11, which lacked *Acetobacter* and was instead dominated by *Gluconobacter, Saccharomyces* and *Ogataea*. Account 4 exhibited the broadest range of detectable genera, indicating a more taxonomically mixed community. In contrast, the other accounts showed narrower community compositions dominated by a few taxa. For example, account 3 was dominated by a smaller number of microorganisms, namely beer-spoilage-relevant genera such as *Acetobacter* and *Pichia*. Further evidence of account-specific variation came from paired beer samples, L1A/L1B as well as A17A/A17B, collected from different accounts (Figures 3B, 4B). Despite being identical products, each pair showed differences in their microbial profiles, driven by shifts in relative abundances of *Acetobacter, Gluconobacter* and *Levilactobacillus*.

**Figure 5:**
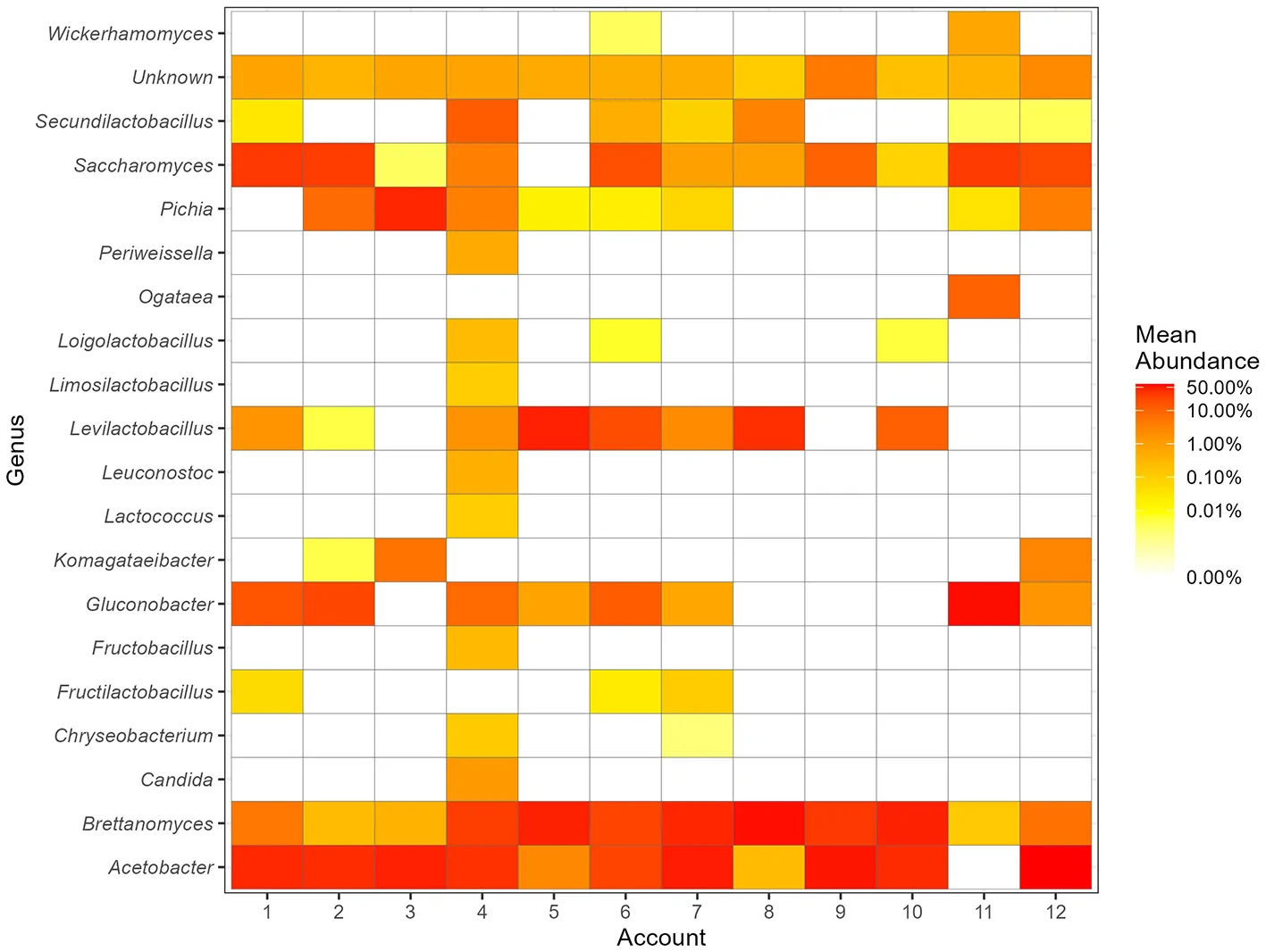
Mean relative abundance of the top twenty genera across accounts. The twenty most abundant genera were identified by calculating the global mean relative abundance across all samples and mean relative abundances were then computed within each account and visualized as a heatmap. Color intensity reflects the mean relative abundance on a logarithmic scale, with warmer colors indicating higher relative abundance.

### 3.3 Microbiome diversity profiles

Previous analyses of the microbiome of beer samples have typically focused on quantification of key species and their relative abundance (Bose et al., 2021; Jevons and Quain, 2022). While informative, this approach does not always allow for a deep understanding of innate variation in complex systems and is of limited value when comparing data obtained across multiple accounts comprising samples from different beer styles. To rectify this, both alpha and beta diversity across the sample set were investigated in this study. Alpha diversity was evaluated by incorporating the Shannon and Simpson diversity indices, which are quantifiable measures of the biodiversity of a specific habitat, focused on rare and dominant species respectively. These account for species richness and species evenness (i.e. how evenly the individuals are distributed among those species). Beta diversity was expressed via Bray-Curtis dissimilarity and reflects how variable samples were from one another in terms of species diversity.

To provide an indication of microbial diversity, alpha diversity metrics were initially applied to describe microbial richness and evenness within accounts (Figure 6). The observed richness reflects the number of unique taxa detected per sample, while the Shannon and Simpson indices incorporate both richness and the relative distribution of taxa. It can be seen that observed richness varied between accounts (Figure 6A), with account 4 exhibiting the highest richness, indicating the presence of a greater number of unique taxa. However, this account also showed the greatest variability among samples, suggesting inconsistent microbiological profiles within the same venue. In contrast accounts 5, 8 and 10 reported the lowest richness values, indicating simpler communities with fewer detected taxa. Analysis of data using the Shannon diversity index (Figure 6B) showed moderate variation across accounts, reflecting differences in both richness and evenness of microbial communities. Accounts with higher Shannon indices, reported in accounts 1, 4 and 6, exhibit more balanced distributions of taxa, whereas lower Shannon diversity, observed in accounts 3, 5, 7 and 8, reflect uneven communities dominated by a small number of taxa. This pattern was reinforced by Simpson diversity (Figure 6C), which showed reduced values in the same accounts, indicating strong dominance by one or a few taxa. Statistical analysis (Kruskal-Wallis tests) found no statistical differences in observed richness (*p* = 0.274), Shannon diversity (*p* = 0.221), or Simpson diversity (*p* = 0.525) between accounts.

**Figure 6:**
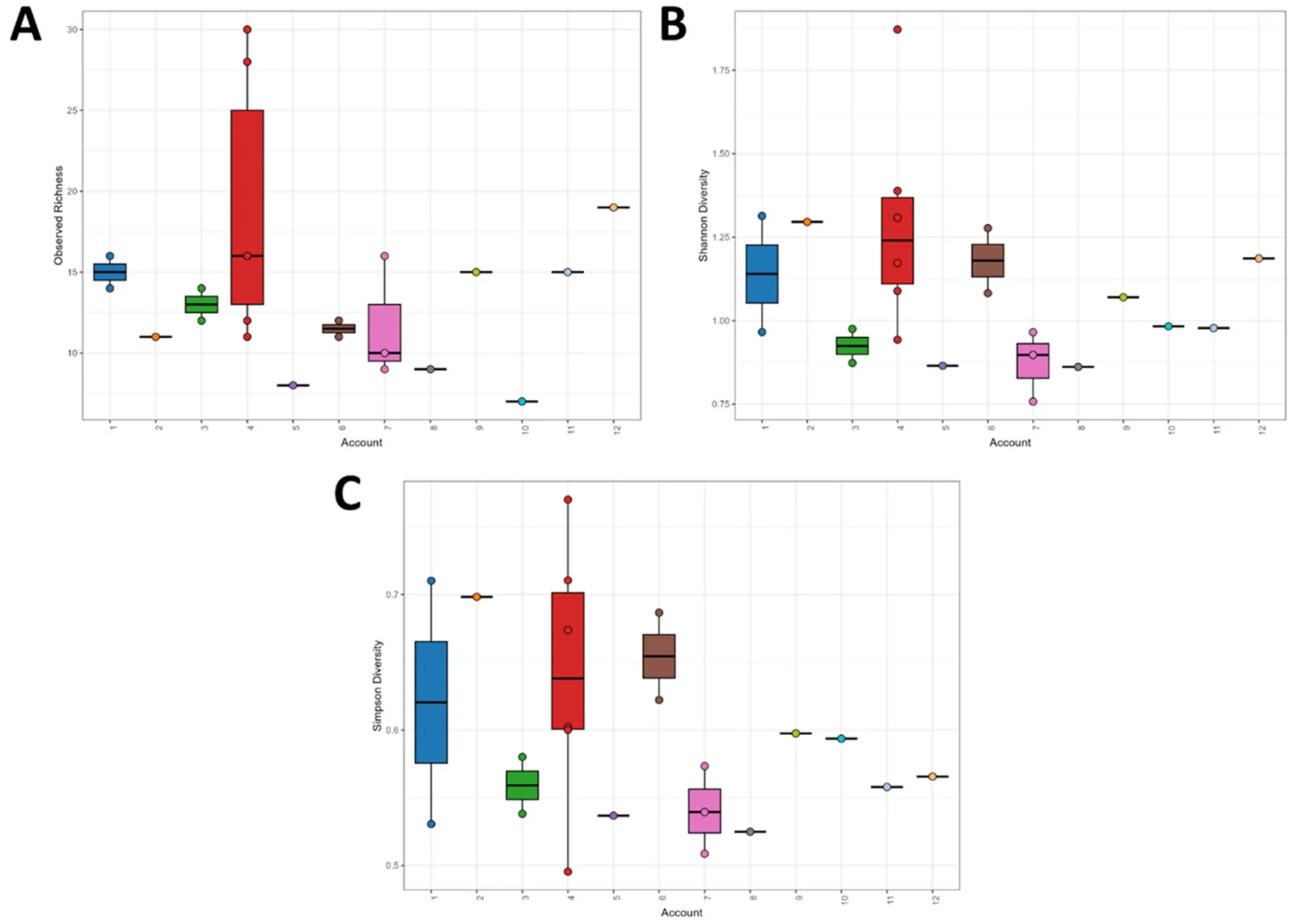
Alpha diversity indices describing species richness and evenness across different accounts. Points represent individual samples, and boxplots show variation within accounts (denoted by the interquartile range). Metrics shown are: **(A)** Observed richness, indicating the total number of taxa detected by account; **(B)** Shannon index, reflecting richness weighted by relative abundances; and **(C)** Simpson index, highlighting dominance/evenness within communities.

To determine variation in microbial biodiversity between samples, beta diversity analysis was performed using Bray-Curtis dissimilarities and visualized by Principal Coordinates Analysis (PCoA; Figure 7). Overall, samples showed separation according to sampled account, although the strength of this pattern and clustering varied between accounts. Samples from some accounts, particularly accounts 3 and 7, clustered closely together, indicating similar microbial community composition. However, this pattern was not consistent across all accounts, for example samples from accounts 1, 4 and 6 were more dispersed in ordination space, suggesting greater within-account variation. Paired samples representing the same beer product collected from different accounts (e.g., L1A/L1B and A17A/A17B) plotted adjacent to one another but did not show tight clustering, indicating that product identity alone did not fully explain microbial community structure. In addition, a PERMANOVA analysis was performed to test whether the concentration of ethanol, beer style, production method and/or account (Table 1) could explain differences in microbial community structure. Ethanol content, beer style and production method did not significantly explain microbial community structure when tested individually (*p* > 0.05), indicating that product-level characteristics were not drivers of microbial community variation. However, a significant proportion of the variation in microbial community composition could be attributed to the sampled account itself, with this explaining 72.6% of variance (*p* = 0.01). Overall, these results suggest that microbial diversity in draught no- and low-alcohol beers is primarily influenced by location, with beers from different accounts often harboring distinct microbial communities. However, not all accounts exhibited tightly clustered microbial communities, and some showed greater within account-variability. This variability may reflect differences in product throughput, or the use of different taps or beer lines within the same account.

**Figure 7:**
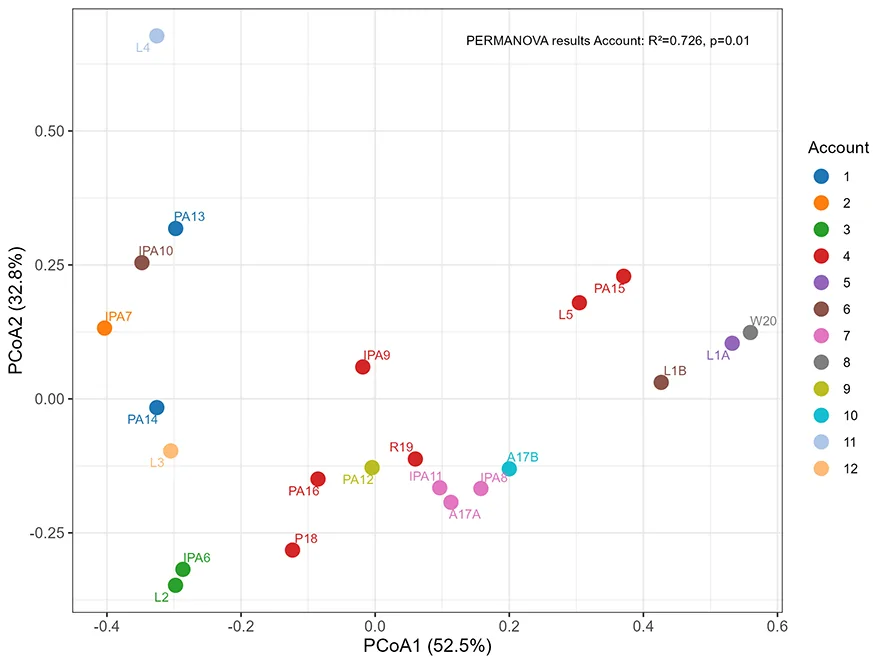
Principal Coordinates Analysis (PCoA) of microbial composition based on Bray-Curtis dissimilarity. Each point represents the microbial profile of an individual beer sample, with accounts indicated by color. A PERMANOVA test was used to evaluate whether microbial composition differed significantly among accounts (*p* < 0.05).

## 4 Discussion

### 4.1 Spoilage-relevant microorganisms

Through screening of NoLo draught beer samples a distinct group of microorganisms emerged as dominant members of the draught no- and low-alcohol beer environment, subsequently classified as core microbiome members. Many of the dominant taxa identified are well-recognized as being recurrent beer spoilage organisms (Paradh, 2015; Powell and Kerruish, 2017; Suzuki, 2015) and have been associated with biofilms in draught dispense systems (Jevons and Quain, 2022). These include *Acetobacter* (Kubizniaková et al., 2021; Thomas and Whitham, 1996), *Lactobacillus* (Walker et al., 2007), *Pediococcus* (Heger and Russell, 2021; Thomas and Whitham, 1996)*, Saccharomyces* and *Brettanomyces* species (Dimopoulou et al., 2019). Species-level resolution further reinforced this interpretation, as dominance patterns were frequently driven by *Brettanomyces anomalus, Brettanomyces bruxellensis, Levilactobacillus brevis* (basionym *Lactobacillus brevis*) and *S. cerevisiae*; all species previously associated with beer spoilage and persistence in brewery or dispense environments (Jevons and Quain, 2022). These findings highlight that many of the dominant microorganisms detected here were not transient contaminants but were capable of persisting within draught dispense systems, irrespective of alcohol content

These findings highlight that many of the dominant microorganisms detected here are not transient contaminants but are capable of persisting within draught dispense systems. Although the focus of this study was on spoilage-relevant microorganisms, no pathogenic microorganisms (such as *Escherichia coli* or *Salmonella* species) were detected in any of the samples analyzed. This is consistent with Rachon et al. (2026), who similarly reported no detection of these microorganisms across 90 draught non-alcoholic beer samples.

It should be noted that the no- and low-alcohol draught beer samples analyzed in this study were subjected to static incubation at 30 °C for 4 days (forcing) to promote the growth of microorganisms capable of growing in beer. It is acknowledged that this approach inherently favors microorganisms that are able to grow under the conditions applied, which inevitably differ from those encountered in draught dispense systems with respect to temperature, oxygen availability and the absence of flow. As a consequence, the sequencing results represent the microbial community following the forcing treatment, and therefore reflect microorganisms capable of surviving and proliferating under these conditions, rather than providing a direct representation of the microbial community present in the beer at the point of sampling. Furthermore, caution should be taken since direct sequencing cannot distinguish between viable and non-viable cells. Although the presence of an unwanted organism (live or dead) is informative, future work incorporating culture-based techniques, viability assays or qPCR may reveal insight into both microbial community composition and functional potential.

#### 4.1.1 Lactic acid bacteria (LAB)

It should be emphasized that the microorganisms identified in this study are not simply indicators of ineffective dispense cleaning, but active drivers of spoilage. Critically, lactic acid bacteria (LAB) represent the most significant Gram-positive spoilage bacteria in brewing, believed to be responsible for 60–70% of all reported cases of beer spoilage (Menz et al., 2010; Rouse and Van Sinderen, 2008; Sakamoto and Konings, 2003). Among LAB, the genera *Lactobacillus* and *Pediococcus* are recognized as key spoilage microorganisms (Rouse and Van Sinderen, 2008). In particular, *Levilactobacillus brevis* (basonym *Lactobacillus*) strains are frequently reported as being the most prevalent most difficult microorganisms to eliminate from the beer environment. This is largely due to their resistance to antimicrobial hop compounds, confered by the hop resistance genes identified on plasmids *horA, horC*, and *hitA* (Bucka-Kolendo et al., 2023; Sakamoto et al., 2001; Suzuki et al., 2006), as well as their ability to form biofilms and persist in brewery environments (Leathers et al., 2014; Rouse and Van Sinderen, 2008). *Secundolactobacillus* (basonym *Lactobacillus*) *paracollinoides* has also been reported as a hard-to-culture beer spoilage LAB species, showing strong spoilage potential comparable to that of *L. brevis* (Suzuki et al., 2008, 2004a,b). Consistent with these reports, *L. brevis* and *S. paracollinoides* were two of the most abundant species detected in the present study. Given the prevalence and recognized spoilage capacity of these microorganisms, their occurrence is concerning; as heterofermentative LAB, they are able to produce acetic acid, ethanol, diacetyl and carbon dioxide in addition to lactic acid. The formation of these metabolites can negatively influence the final beer product due to acidification, turbidity, production of buttery off-flavors and gushing (Bergsveinson and Ziola, 2017; Pfeiler and Klaenhammer, 2007).

#### 4.1.2 Acetic acid bacteria (AAB)

Acetic acid bacteria (AAB) are Gram-negative aerobic bacteria frequently associated with draught beer, where the presence of oxygen and elevated temperatures can favor their growth (Storgårds, 2000). Among AAB, the genera *Acetobacter* and *Gluconobacter* are most commonly implicated in beer spoilage. These bacteria are able to tolerate acidic conditions, metabolize a broad range of substrates, and oxidize ethanol to acetic acid, resulting in an unpleasant vinegary off-flavor in beer and deacidification (Kubizniaková et al., 2021; Paradh, 2015). Growth of *Gluconobacter* can also lead to haze formation, pellicle development, and in some strains, ropiness caused by the production of dextrans and levans which increase beer viscosity (Hornsey, 1999). Across the samples analyzed in this study, 28 species of *Acetobacter*, nine species of *Gluconobacter* and two species of *Gluconoacetobacter* were detected. Notably, the most abundant *Acetobacter* species identified, *A. oryzoeni* and *A. sicerae* (detected in 20 and 18 beer samples, respectively), have not previously been associated with beer. Both species are usually more closely linked to sugar-rich fermentation environments. For example, *A. oryzoeni* has previously been isolated from Korean rice wine vinegar (Baek et al., 2020) and palm wine fermentations (Attchelouwa et al., 2025). However, comparative analysis based on 16SrRNA gene sequence similarity has indicated that there is a close relationship between this organism and *A. pasteurianus* (LMG1262), a well-established brewery spoilage microbe (Kubizniaková et al., 2021). Similarly, *A. sicerae* has previously been associated with cider contamination (Shimwell and Carr, 1958) and in spoiled kefir made using sugar syrups (Li et al., 2014). The ability of these species to utilize fructose, sorbitol and glycerol as single carbon sources suggests effective adaptation to high-sugar niches which may confer a selective advantage in no- and low-alcohol beers where residual fermentable sugars can sometimes be present, either due to the method of production (specifically arrested fermentation) or through post-production supplementation with sugar syrups. This provides support to previous suggestions that NoLo beers may be vulnerable to microorganisms that are not traditionally associated with beer, including those that are adapted to sugar-rich, low-ethanol environments (Roselli et al., 2024).

#### 4.1.3 Beer-spoiling yeast

*Saccharomyces* and *Brettanomyces* species are recognized as being among the primary yeasts responsible for spoilage of standard alcoholic beer types (Suiker and Wösten, 2022). In this study, *Brettanomyces* yeast (specifically *B. anomalus* and *B. bruxellensis*), were the most prevalent organism identified overall; these strains act to produce acetic acid and a range of volatile phenolic compounds including 4-ethylguiaiacol (4-EG) and 4-ethylphenol (4-EP), formed via the decarboxylation of hydroxycinnamic acids (Chatonnet et al., 1992; Ryder et al., 1978). These are responsible for characteristic ‘phenolic off-flavour' (POF), often described as medicinal, spicey, smokey, barnyard or clove- like (Thurston and Tubb, 1981). In addition, *Brettanomyces* species can synthesize fatty acids, which negatively affect beer foam stability (Bamforth, 1985; Olšovská et al., 2019; Wilde et al., 2003) and can impart rancid, cheesy, soapy or fatty aroma characteristics. *B. bruxellensis* was identified as the most abundant species found across all NoLo samples, in line with previous reports suggesting that this species is the most frequently encountered in spoilage of standard beer types (Crauwels et al., 2014). It should be noted that multiple studies have revealed a high degree of phenotypic diversity within *B. bruxellensis* yeast (Avramova et al., 2018), especially with respect to sugar metabolism (Conterno et al., 2006; Crauwels et al., 2015, 2017), nitrogen source utilization (Borneman et al., 2014; Crauwels et al., 2015) and abiotic factors including temperature and pH preference (Blomqvist et al., 2010; Conterno et al., 2006), oxygen availability (Aguilar Uscanga et al., 2003; Capusoni et al., 2016; Du Toit et al., 2005) and sensitivity to sulfur dioxide (Agnolucci et al., 2010; Barata et al., 2008; Conterno et al., 2006; Crauwels et al., 2017; Curtin et al., 2012; Du Toit et al., 2005; Vigentini et al., 2013). This phenotypic plasticity indicates that *B. bruxellensis* is likely to remain a major concern for draught no- and low-alcohol beer quality.

Although not as prevalent as *Brettanomyces* yeast in this instance, *Saccharomyces* yeast were also evident. Non-domesticated *Saccharomyces* strains are often considered to be the most problematic yeast contaminants in beer, primarily because they can be difficult to detect due to their phenotypic and genetic similarity with production strains. Inevitably, the broad category of ‘*Saccharomyces* yeast' also includes diastatic subspecies of *S. cerevisiae* (sometimes referred to as *S. cerevisiae* var. *diastaticus*), which are characterized simply by the presence of the STA1 (glucoamylase) gene (Adam et al., 2004; Tamaki, 1978) and cannot be differentiated based on the ITS region alone. While the majority of beer-spoiling yeast cause haze formation and the production of off-flavors (Suiker and Wösten, 2022), diastatic strains are particularly adept at utilizing residual carbohydrates, including dextrins and starch, leading to super attenuation, overcarbonation and a weakened ‘body' (Andrews and Gilliland, 1952; Hutzler et al., 2012). Historically, diastatic microorganisms have been most problematic in small-pack beers, where elevated carbon dioxide levels can lead to bombages or exploding bottles (Jevons and Quain, 2022). However, in draught no- and low-alcohol beers, diastatic yeast may pose an emerging spoilage risk, as both the yeast itself and its ascospores are able to readily survive in alcohol free beer (Suiker et al., 2021). Since NoLo beers can contain higher residual extracts than standard beer types, uncontrolled fermentation post-production can increase ethanol concentrations beyond legal limits, with potential quality and legislative implications (Roselli et al., 2026).

Although *S. cerevisiae* strains were the most predominant *Saccharomyces* yeast found, *Saccharomyces uvarum* was also encountered, with the potential to cause similar affects in the final product. This yeast is more commonly associated with wine and cider fermentations rather than beer production or spoilage, but was detected at relatively high abundance (identified across 12 samples), supporting previous observations in alcoholic draught beer (Jevons and Quain, 2022). *S. uvarum* is typically cryotolerant (Gamero et al., 2013; García-Ríos et al., 2016), a trait that may contribute to its presence in UK draught beer systems, which are typically maintained at 2–12 °C.

The widespread presence of beer-spoiling yeasts may be facilitated by their association with biofilms (Timke et al., 2008). It has previously been reported that *S. diastaticus* and *B. bruxellensis* species can be found within biofilms isolated from brewery environments, alongside yeasts such as *Candida* and *Pichia* (Suiker and Wösten, 2022). Although *Candida* yeast were not especially prevalent in this study, *Pichia* yeast were commonly encountered. It has been suggested that in brewery environments, surface colonization can be initiated by key Gram negative bacteria, including *Pseudomonas* spp., followed by adhesion of yeasts such as *Pichia anomala*, as well as *Candida sake* and *Debaryomyces hansenii*. Subsequent colonization by *S. diastaticus* and/or *B. bruxellensis* can then result in the formation of biofilms with significant spoilage potenitial (Suiker and Wösten, 2022; Timke et al., 2008). In the present study, 16 *Pichia* species (with *P. membranifaciens* and *P. manshurica* being the most abundant) and four *Candida* species were detected, along with *Wickerhamomyces anomalus* (formerly *Pichia anomala*). The presence of multiple *Pichia* and *Candida* species lends support to the theory that draught dispense systems may provide suitable conditions for the establishment of mixed-species biofilms associated with beer spoilage. Given that microorganisms within biofilms exhibit increased resistant to antibiotics, disinfectants, UV light and desiccation (Quain and Storgårds, 2009; Storgårds et al., 2006), the importance of rigorous draught line hygiene and the implementation of cleaning regimes to minimize spoilage risk in no- and low-alcohol beers should be emphasized.

### 4.2 Account-level factors drive microbial variability

The data presented here supports previous observations that the draught no- and low-alcohol beer microbiome is shaped not only by the beer itself but by account-specific factors, including dispense environment and hygiene practices (Roselli et al., 2026). Although alpha diversity metrics indicated limited differences between accounts, beta diversity analysis revealed pronounced shifts in community composition. The differences in dominant genera observed across accounts demonstrate that although spoilage-associated genera (*Acetobacter, Brettanomyces, Gluconobacter, Levilactobacillus, Pichia* and *Saccharomyces*) were widespread across accounts, the relative abundances and microbial load varied considerably between venues. For example, some accounts harbored complex, relatively balanced communities, whereas others were dominated by a small number of high-abundance taxa. Principal Coordinates Analysis (PCoA) of Bray-Curtis dissimilarities further highlighted that samples clustered primarily by account, supporting the notion that venue-specific factors exert a strong influence on microbiome composition. Paired samples of the same beer collected from different accounts (e.g., L1A/L1B and A17A/A17B) were found to be placed adjacent to one another but did not show tight clustering, with differences driven by shifts in relative abundances of *Acetobacter, Gluconobacter* and *Levilactobacillus*. These findings indicate that, although the beer matrix contributes to microbial community composition, account-specific factors introduce measurable variation in draught no- and low-alcohol beer microbiomes. This is consistent with Bose et al. (2021), who reported that microbial communities recovered from retail draught beer lines changed over the course of a year and that beer chemistry alone did not fully determine community structure. A similar principle was demonstrated by Bokulich et al. (2015), who showed that microbial communities in brewery environments were spatially structured, with beer-exposed sites associated with a greater abundance of spoilage-related genes. Whilst this was not investigated in the current study, Bokulich et al. (2015) also reported certain seasonal changes in brewery-resident microbial communities. Together, these studies support the theory that beer-associated environments can act as reservoirs for spoilage microorganisms and that microbial communities are dynamic and shaped by environmental and operational factors, rather than by beer matrix alone. Therefore, account-specific patterns observed in the present study are likely to reflect differences in the microbial communities established within individual dispense systems, including biofilms present in beer lines, taps or other contact surfaces. These site-specific reservoirs could help explain the high variability observed between accounts, although direct sampling of multiple locations within each site over time would be required to confirm this.

## 5 Conclusions

This study provides the first amplicon-sequencing based characterization of the microbial communities present in draught no- and low-alcohol beers. Using this methodology, a broad and comprehensive analysis of the microbiome could be achieved, including identification of species that are difficult to detect using traditional culture-based approaches. Across samples, species belonging to *Acetobacter, Brettanomyces* and *Saccharomyces* consistently appeared as core members of the draught no- and low-alcohol beer microbiome. Microbial communities clustered strongly by account, indicating that practices such as draught line hygiene, cleaning frequency and handling practices are key drivers of contamination patterns in the on-trade. These findings highlight the need for rigorous hygienic handling of draught no and low-alcohol products, particularly within long-line dispense systems. It is anticipated that tailored cleaning protocols, along with consistent and effective hygiene maintenance practices, are likely to play a critical role in reducing the risk of microbial contamination in NoLo beer, ensuring both product quality and consumer satisfaction.

## Data Availability

The datasets presented in this study can be found in online repositories. The names of the repository/repositories and accession number(s) can be found at: https://www.ebi.ac.uk/ena, PRJEB123733.

## Footnotes

1 The precise definition of a no- or low- alcohol (NoLo) beer is not universal, and the defining values with respect to alcohol content vary between Europe, United States and elsewhere (Quain, 2021). For the purpose of this article the terminology used will refer to UK legislation; no-alcohol beers are labeled as containing ≤ 0.05 % ABV and low-alcohol beers ≤ 1.2 % ABV (GOV.UK, 2018).
