# Supplementary material for: The microbiome of draught no- and low-alcohol beer
Source: Front Microbiol. 2026 Oct 28;17:1966818. doi: 10.3389/fmicb.2026.1966818 (PMC13752092; doi:10.3389/fmicb.2026.1966818)
Supplement: Supplementary file 1 [file Table_1.docx]

**Supplementary Table 1.** Summary of sequencing run quality metrics for all amplicons, including read quality, read length statistics, base yield above read-length thresholds and read counts.

| Sample | Read quality | | Read length | | | | | | Base yield above read length | | Number of reads | |
| --- | --- | --- | --- | --- | --- | --- | --- | --- | --- | --- | --- | --- |
|  | **Mean** | **Median** | **Mean** | **Median** | | **Min** | | **Max** | **Total yield (Gb)** | **N50 (kb)** | **Reads** | **Unclassified (%)** |
| L1A | 15.6 | 15 | 2060.4 | 1659 | 108 | | 12274 | | 0.532 | 108 | 257956 | 0.56 |
| L1B | 15.6 | 15 | 2023.2 | 1656 | 113 | | 10165 | | 0.636 | 113 | 314275 | 0.49 |
| L2 | 15.5 | 15 | 1983 | 1579 | 115 | | 10257 | | 0.575 | 115 | 290126 | 1.12 |
| L3 | 15.4 | 15 | 1897.1 | 1576 | 115 | | 12729 | | 0.627 | 115 | 330264 | 2.3 |
| L4 | 15.7 | 15 | 2082.8 | 1573 | 117 | | 11160 | | 0.727 | 117 | 349143 | 0.36 |
| L5 | 15.4 | 15 | 1892.6 | 1650 | 109 | | 8286 | | 0.563 | 109 | 297302 | 0.28 |
| IPA6 | 15.5 | 15 | 2027.1 | 1571 | 117 | | 11014 | | 0.77 | 117 | 379602 | 0.32 |
| IPA7 | 15.5 | 15 | 2003.6 | 1576 | 109 | | 10494 | | 0.76 | 109 | 379183 | 0.35 |
| IPA8 | 15.5 | 15 | 1998.4 | 1585 | 111 | | 13675 | | 0.562 | 111 | 281313 | 1.33 |
| IPA9 | 15.4 | 15 | 1912.4 | 1575 | 116 | | 10681 | | 0.667 | 116 | 348979 | 0.92 |
| IPA10 | 15.7 | 15 | 2063.3 | 1577 | 119 | | 13723 | | 0.79 | 119 | 383080 | 0.54 |
| IPA11 | 15.6 | 15 | 2029.5 | 1581 | 118 | | 9820 | | 0.907 | 118 | 446996 | 0.14 |
| PA12 | 15.4 | 15 | 2011.2 | 1580 | 115 | | 15240 | | 0.836 | 115 | 415750 | 4.43 |
| PA13 | 15.6 | 15 | 2099.4 | 1576 | 120 | | 20140 | | 0.629 | 120 | 299844 | 0.56 |
| PA14 | 15.7 | 15 | 1998.6 | 1576 | 119 | | 10999 | | 0.463 | 119 | 231539 | 0.93 |
| PA15 | 15.7 | 15 | 2018.7 | 1654 | 111 | | 8614 | | 0.502 | 111 | 248639 | 0.06 |
| PA16 | 15.4 | 15 | 1842.7 | 1575 | 115 | | 9428 | | 0.558 | 115 | 302647 | 0.28 |
| A17A | 15.5 | 15 | 1987.1 | 1580 | 111 | | 13681 | | 0.67 | 111 | 337062 | 0.19 |
| A17B | 15.6 | 15 | 2026.3 | 1647 | 110 | | 7173 | | 0.685 | 110 | 337984 | 0.2 |
| P18 | 15.2 | 15 | 1858 | 1576 | 119 | | 14210 | | 0.587 | 119 | 316030 | 2.7 |
| R19 | 15.4 | 15 | 1933.5 | 1577 | 115 | | 10213 | | 0.729 | 115 | 376936 | 0.37 |
| W20 | 15.6 | 15 | 2157.8 | 2663 | 114 | | 8979 | | 0.684 | 114 | 317176 | 0.11 |
